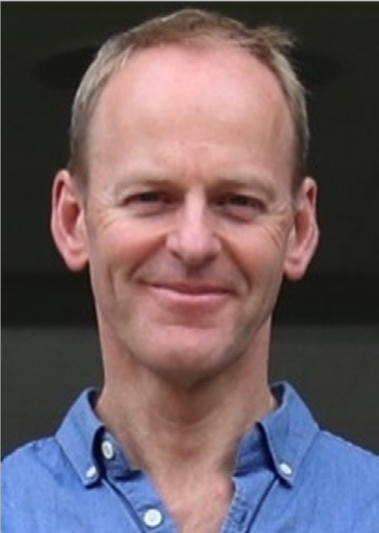# Enzymatic Kinetic Resolution by Addition of Oxygen

**DOI:** 10.1002/anie.202011468

**Published:** 2020-12-22

**Authors:** Lucy A. Harwood, Luet L. Wong, Jeremy Robertson

**Affiliations:** ^1^ Department of Chemistry University of Oxford Chemistry Research Laboratory Mansfield Road Oxford OX1 3TA UK; ^2^ Department of Chemistry University of Oxford Inorganic Chemistry Laboratory South Parks Road Oxford OX1 3QR UK; ^3^ Oxford Suzhou Centre for Advanced Research Ruo Shui Road, Suzhou Industrial Park Jiangsu 215123 P. R. China

**Keywords:** biocatalysis, kinetic resolution, oxidative enzymes, total synthesis

## Abstract

Kinetic resolution using biocatalysis has proven to be an excellent complementary technique to traditional asymmetric catalysis for the production of enantioenriched compounds. Resolution using oxidative enzymes produces valuable oxygenated structures for use in synthetic route development. This Minireview focuses on enzymes which catalyse the insertion of an oxygen atom into the substrate and, in so doing, can achieve oxidative kinetic resolution. The Baeyer–Villiger rearrangement, epoxidation, and hydroxylation are included, and biological advancements in enzyme development, and applications of these key enantioenriched intermediates in natural product synthesis are discussed.

## Graphical abstract

Enzymatic oxidative kinetic resolutions (OKR), in which a new oxygen atom is inserted into the substrate, are highly effective for the synthesis of enantioenriched oxygen‐containing compounds. This Minireview discusses selected enzyme classes that can achieve OKR, biological advancements and protein development, and the use of these key enantioenriched intermediates in natural product synthesis.

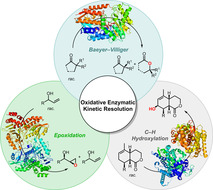

## 1 Introduction

Since Pasteur first reported the separation of the enantiomers of tartaric acid via the sodium ammonium and sodium potassium *rac*‐tartrates in 1848,^[1]^ the resolution of racemic starting materials has been an important method for the production of valuable chiral compounds.^[2]^ Advances in asymmetric catalysis have led to many elegant methods for the synthesis of enantioenriched building blocks, but kinetic resolution remains a key strategy which is widely used in both academia and industry.

This Minireview aims to complement existing reviews on the resolution of racemic substrates^[3]^ by focusing on recent developments (2010–2020) in enzymatic kinetic resolution involving addition of oxygen. Oxidative kinetic resolution (OKR) processes in which there is no addition of oxygen (for example, the use of alcohol dehydrogenases/ketoreductases),^[4]^ or in which addition of oxygen occurs as a result of hydrolysis (such as epoxide hydrolases),^[5]^ and enzymatic desymmetrisation^[6]^ are outside of the scope of this review. We aim instead to highlight the value of using enzymatic oxidation for the kinetic resolution of racemic substrates as a complementary technique to traditional chemical methods.

**Figure 1:**
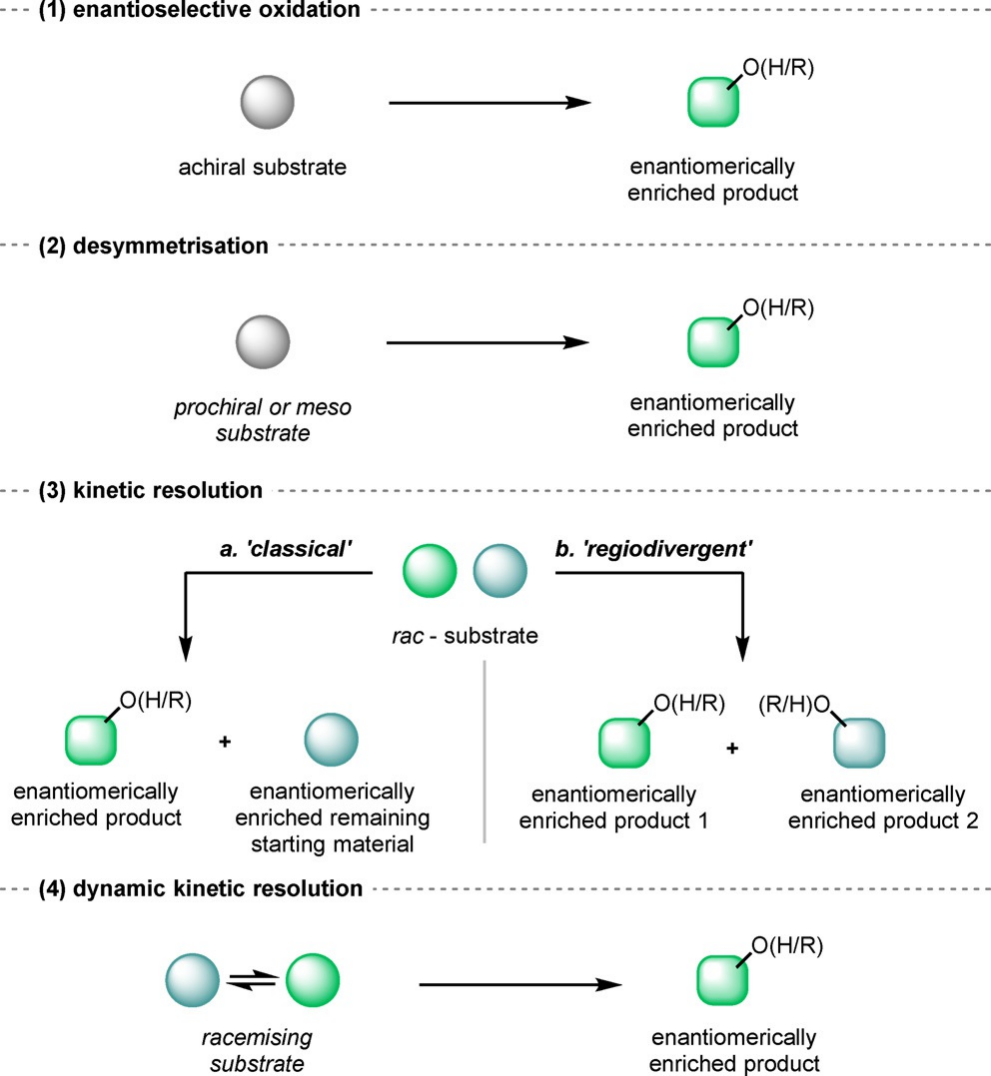
Strategies for the production of enantioenriched compounds via addition of oxygen.

The sp^3^‐carbon‐bound hydroxyl group is arguably the most important and versatile functional group inroad to the assembly of small organic molecules, by virtue of: 1) the possibility of innate stereochemistry at the carbinol centre and well‐developed stereospecific reactions thereof; 2) oxidation to a ketone that links to the vast chemistry of the carbonyl group including, inter alia, olefination, reductive amination, oxidative ring‐expansion, α‐alkylation, and 1,2‐addition; 3) enabling O‐tethered reactions, including C–H insertion processes; 4) enabling O‐directed reactions; and 5) acylation and sulfonylation chemistry; and so on. As a result of this central role in synthesis, multiple strategies have been devised for accessing enantiomerically enriched or enantiomerically pure (collectively described herein as enantioenriched) oxygenated compounds (Figure 1), including:

1. *Enantioselective oxidation*.^[7]^ The use of an achiral substrate in combination with a chiral reagent/catalyst to achieve an enantioselective addition of oxygen.
2. *Desymmetrisation*.^[8]^ A subset of (1) when the achiral starting material is a prochiral or meso substrate, where a symmetry element in the substrate is removed and chirality is introduced, providing up to 100 % maximum yield of enantioenriched chiral compounds.
3. a. *Classical kinetic resolution (KR)*. The selective addition of oxygen to one enantiomer of a racemic substrate as a result of a difference in the reaction rates of the enantiomers; where the enantiomers do not interconvert, the reaction is limited to a 50 % yield of the product. Nevertheless, high enantioselectivities of both product and unreacted starting material are possible. Successful KR is usually characterised using Chen's method of calculating E value.^[9]^
  b. *Divergent reaction on a racemic mixture (divergent RRM)*. The enantiomers of a racemic substrate are oxidised by the same reagent producing distinct enantioenriched products. Parallel kinetic resolution (PKR) is a subset of divergent RRM, where two distinct chiral reagents react with each enantiomer differently to produce two non‐enantiomeric products.^[10]^
4. *Dynamic kinetic resolution (DKR)*. The substrate is able to racemise, either spontaneously or via an additional chemical reagent or enzyme, and one enantiomer is selectively oxidised. For an effective DKR the rate of racemisation must be faster than the rate of oxidation of the unwanted enantiomer. A quantitative yield of product is theoretically possible, thereby overcoming a limitation of classical kinetic resolution.

A recent resurgence in applications of biocatalysis for asymmetric synthesis^[11]^ has been driven in part by vast improvements in protein engineering techniques, including but not limited to computation, improved technical protocols, and directed evolution. Analogous to the design and optimisation of chemical catalysts, protein engineering has enabled the design of bespoke enzymes to address unique synthetic challenges. For many industrial processes, enzymatic methods may provide attractive alternatives to equivalent chemical reactions as a result of their generally mild reaction conditions, often green and sustainable processes, with less hazardous reagents, producing less toxic waste and by‐products, and reduced costs on large scale.^[12]^ For example, lipase‐mediated partial hydrolysis has been a long‐standing and dominant strategy for enzymatic resolution that is widespread in both academic and industrial route development.^[13]^

Enzymes in Nature often evolve to catalyse reactions of specific substrates or specific substrate classes, which is advantageous for their application to KR. Within either the wild‐type, or variants bearing specific mutations, an enzyme's active site, by providing an inherently chiral environment, can achieve highly enantiodiscriminating catalysis, resulting in effective resolutions when presented with racemic substrates. To the enzyme, the substrate enantiomers are totally different molecules whose mirror‐image relationship is incidental. Oxygenases, often using molecular dioxygen as their [O] source, catalyse the addition of oxygen to an organic compound either chemo‐, regio‐, or enantioselectively to yield valuable oxygenated products. Biocatalytic oxidation is becoming accepted as a key component in the mainstream organic chemist's toolbox, perhaps still being employed only after classical chemistry has failed, despite such enzymatic methods allowing access to more efficient and elegant syntheses.^[14]^

It is not always viable (practically or economically) to use asymmetric catalysis or other modes of enantioselective synthesis in a synthetic route, and often the racemic synthesis followed by resolution at some point is the superior solution. This Minireview aims to highlight the benefits of using oxidising enzymes for kinetic resolution and stimulate synthetic chemists to include this approach in new syntheses and thereby advance this developing field.

**Scheme 1:**
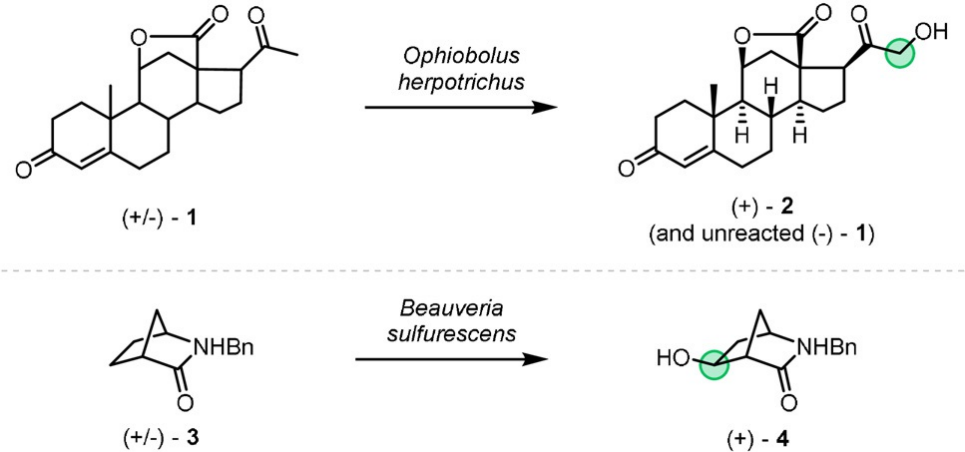
Representative early examples of enzymatic OKR.

Early reports of enzymatic transformations that involve addition of oxygen were typically of whole‐cell microbial processes.^[15]^ Notable examples include a 1956 total synthesis of the steroid (+)‐aldosterone, where a key racemic intermediate **1** was resolved during hydroxylation by the mould *Ophiobolus herpotrichus* to produce the optically active (+)‐enantiomer of the oxygenated product **2**, leaving the (−)‐enantiomer of the substrate intact (Scheme 1).^[16]^ Furthermore, a report from 1984 described the biohydroxylation of a racemic 2‐azabicyclo[2.2.1]heptane derivative **3** by *Beauveria sulfurescens* that occurs selectively at the 5‐exo C−H bond, producing enantioenriched hydroxylactam (+)‐**4**.^[17]^

## 2 Kinetic Resolution by Selective Action at a Specific Functional Group

### 2.1 Baeyer–Villiger Rearrangement

The flavin‐dependent Baeyer–Villiger monooxygenase (BVMO) catalyses the oxidation of both cyclic and acyclic ketones to lactones and esters, respectively.^[18]^ Traditional chemical methods to achieve the Baeyer–Villiger (BV) transformation^[19]^ typically employ stoichiometric oxidants containing the peroxy linkage; such reagents are often heat‐ and shock‐sensitive, toxic, and expensive, all of which severely constrain their large‐scale industrial application.^[20]^ BVMOs harness aerial molecular oxygen to produce the reactive oxidant in situ at low concentration, offering a safer, green alternative with considerable effort now being made to expand the traditionally limited substrate scope of these enzymes and to improve selectivity.^[21]^

**Scheme 2:**
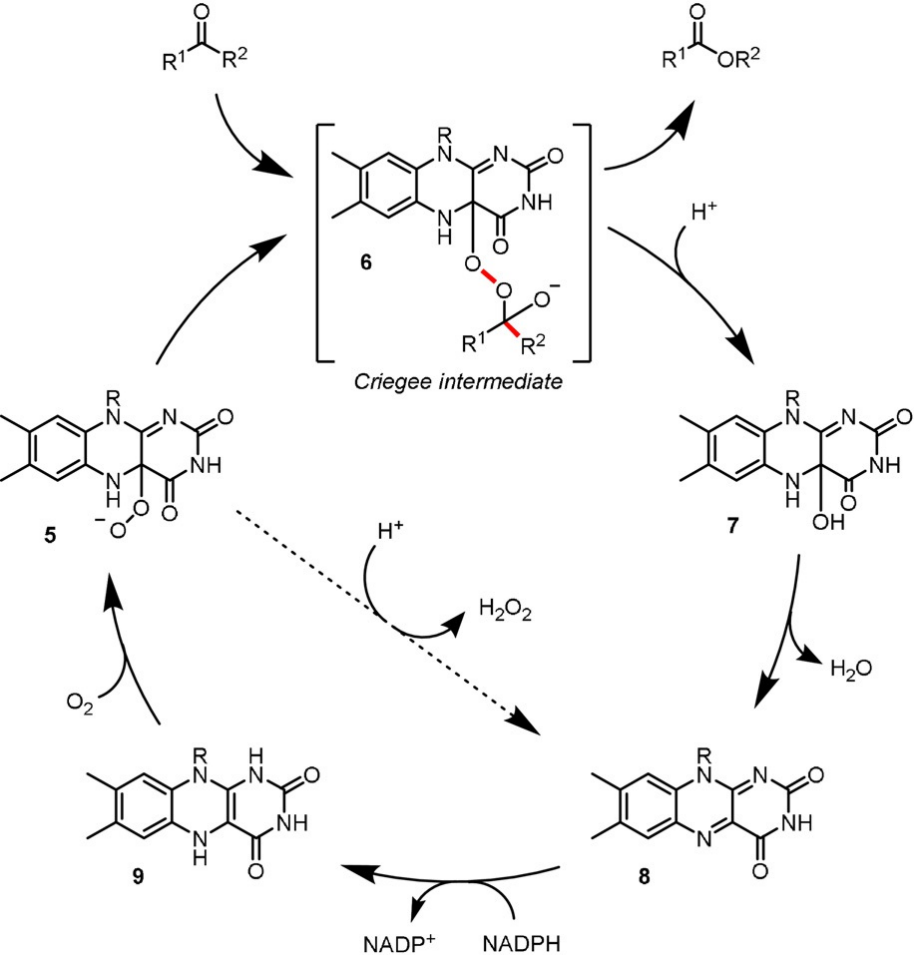
Catalytic cycle for flavin‐dependent BVMO. The anti‐periplanar alignment of the C−C bond and the O−O peroxy bond which is required for bond migration is highlighted in red. The dashed arrow indicates a decomposition pathway of non‐substrate bound peroxyflavin, generating hydrogen peroxide and oxidised flavin **8**.

The catalytic cycle is initiated with the binding of NADPH and subsequent flavin reduction via hydride transfer from the nicotinamide cofactor (Scheme 2). In contrast to acid‐catalysed chemical BV reactions, the catalytically active species here is the deprotonated peroxyflavin **5**. In all monooxygenases there is an “uncoupling” decomposition pathway, where non‐substrate bound peroxyflavin undergoes proton transfer to produce hydrogen peroxide and regenerate flavin. In BVMOs, NADP^+^ has been shown to be essential for stabilisation of this deprotonated intermediate which, upon nucleophilic attack onto the carbonyl of the substrate, forms the key tetrahedral “Criegee intermediate” **6**. It is crucial for this intermediate to achieve an anti‐periplanar alignment of the migrating C−C bond with the peroxy O−O bond to enable bond migration and, with certain substrates, also with an oxygen lone pair.^[22]^ The regioselectivity is subject to influence by multiple subtle factors; nevertheless, it is generally predictable, forming product by migration of the more substituted bond of the substrate. Dehydration of the hydroxylated flavin **7** regenerates flavin **8** to complete the catalytic cycle.

**Figure 2:**
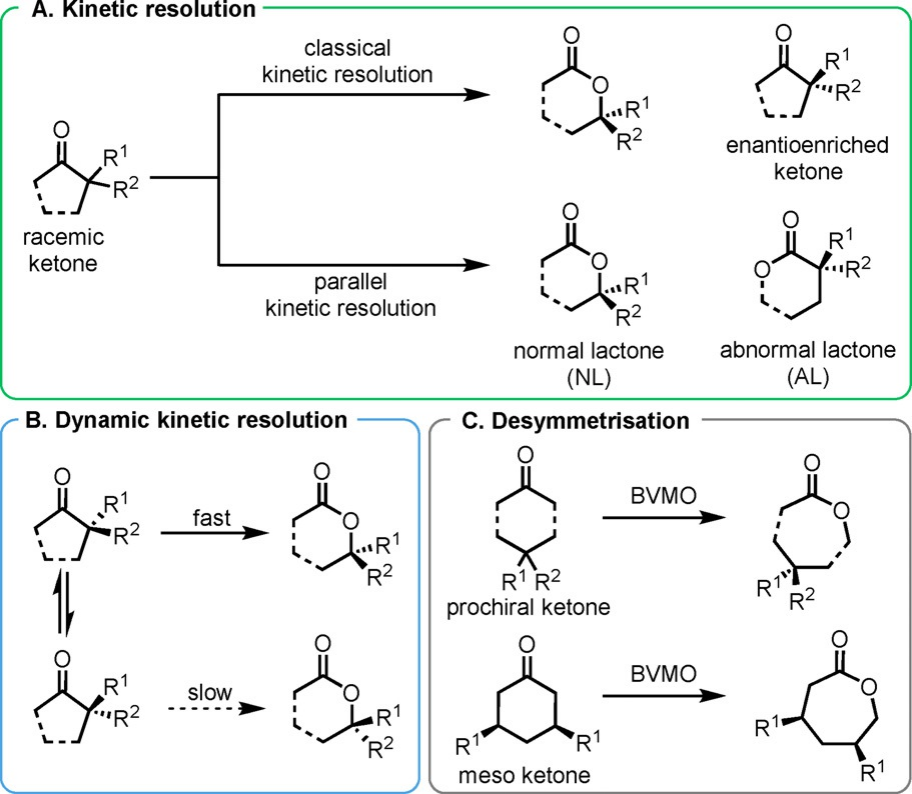
Strategies for the production of enantioenriched lactones by means of BV reaction.

In general, the reaction creates no new stereogenic centres and therefore is only enantioselective through KR of racemic starting materials. Desymmetrisation reactions of achiral precursors (Figure 2 C) are exceptions to this but fall outside the scope of this review. Such processes can be of the “classical kinetic resolution” type (Figure 2 A) where one enantiomer is oxidised to the lactone leaving the remaining substrate enantioenriched, or of the “parallel kinetic resolution” type where each enantiomer gives rise to a different lactone in an enantiodivergent process. If the starting material is able to be racemised, it is possible to achieve a DKR where faster oxidation of one ketone enantiomer can produce up to 100 % yield of enantioenriched lactone (Figure 2 B).

#### 2.1.1 Methodology Overview

Different BVMO subclasses have inherent advantages or disadvantages. For example, cyclohexanone monooxygenase (CHMO) is widely used because of the large substrate scope but its reactions can be challenging to scale up due to thermal instability, whereas phenylacetone monooxygenase (PAMO) is generally more thermally stable but has relatively limited substrate tolerance. Attempts have been made to incorporate the excellent activity of CHMO into PAMO in order to deliver the best characteristics of both prototype enzymes.^[23]^

**Scheme 3:**
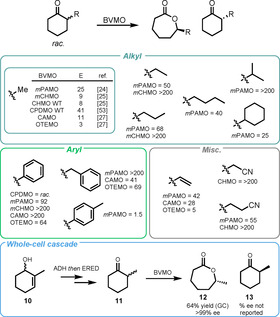
Scope of mono‐α‐substituted cyclohexanone substrates accepted for a range of reported BVMOs. The reported absolute configurations and *ee* values for individual products vary between references.

Cyclohexanones are well‐tolerated substrates for a variety of BVMO subclasses, and there has been considerable research focused on their transformation to *ϵ*‐lactones. The Reetz group expanded the substrate scope of PAMO considerably by using directed evolution to identify, from the X‐ray crystal structure, two distal residues which were able to promote allosteric effects in the shape of the binding pocket of PAMO.^[24]^ This Q93N/P94D variant was shown to catalyse the KR of a wide range of α‐substituted cyclohexanones previously not accepted by WT PAMO (Scheme 3).

BVMO instability has been attributed to unwanted oxidation of sulfur‐containing residues caused by H_2_O_2_ built up from the decomposition pathway of non‐substrate bound BVMO. Reetz described how both the thermal and oxidative stability of a CHMO could be improved by substituting distal amino acid residues Met/Cys for non‐sulfur containing amino acids which are less easily oxidised.^[25]^ These new CHMO variants were able to retain up to 40 % activity after incubation with H_2_O_2_. These oxidation‐resistant mutants showed comparable activity and selectivity compared to WT CHMO for a range of aryl and alkyl substituents (Scheme 3). In a contrasting approach, Bornscheuer reported the use of computational methods to develop CHMO mutants with new disulfide bonds that showed improved oxidative stability with no decrease in performance for the KR of 2‐methylcyclohexanone.^[26]^

Rudroff reported an in‐depth substrate profiling for two new BVMOs: a camphor monooxygenase (CAMO) belonging to the CHMO subgroup and a 2‐oxo‐Δ^3^‐4,5,5‐trimethylcyclopentenyl‐acetyl‐coenzyme A monooxygenase (OTEMO).^[27]^ Unsurprisingly, CAMO showed higher E values for a range of cyclohexanones; however, both enzymes gave generally poor selectivity with *E*=3–69, apart from the substrate with R=Ph which gave *E*>200 (Scheme 3). However, both CAMO and OTEMO were shown to be excellent catalysts for the desymmetrisation of 3‐vinylcyclobutanone and this led to the first chemoenzymatic synthesis of the Taniguchi lactone, a key intermediate in the synthesis of multiple natural products. In a recent report, the Rudroff group was able to use the genomic sequence of a recently published and thermostable BVMO, TmCHMO, for database screening and identified a new BVMO from *Amycolaptosis thermoflava*.^[28]^ This new thermostable monooxygenase was able to process a range of cyclic ketones with excellent conversion and enantioselectivity.

Bornscheuer and Mihovilovic achieved an impressive whole‐cell biocatalytic cascade incorporating the BVMO‐catalysed KR of racemic 2‐methyl cyclohexanone **11** (Scheme 3).^[29]^ Expressing a sequence of alcohol dehydrogenase (ADH), enoate reductase (ERED), and BVMO in *E. coli* allowed the transformation of alcohol **10** through to the corresponding caprolactone **12** in 62 % overall yield and >99 % *ee*. They further developed this cascade by creating a fusion protein of the ERED and BVMO, achieving a 40 % increase in lactone production compared to that achieved with the separate enzymes.^[30]^

Enantioenriched methyl‐substituted lactones produced enzymatically from CHMO have been used as monomers in lipase‐catalysed oligomerisation reactions to form homochiral polyesters with potential applications in material science.^[31]^

**Scheme 4:**
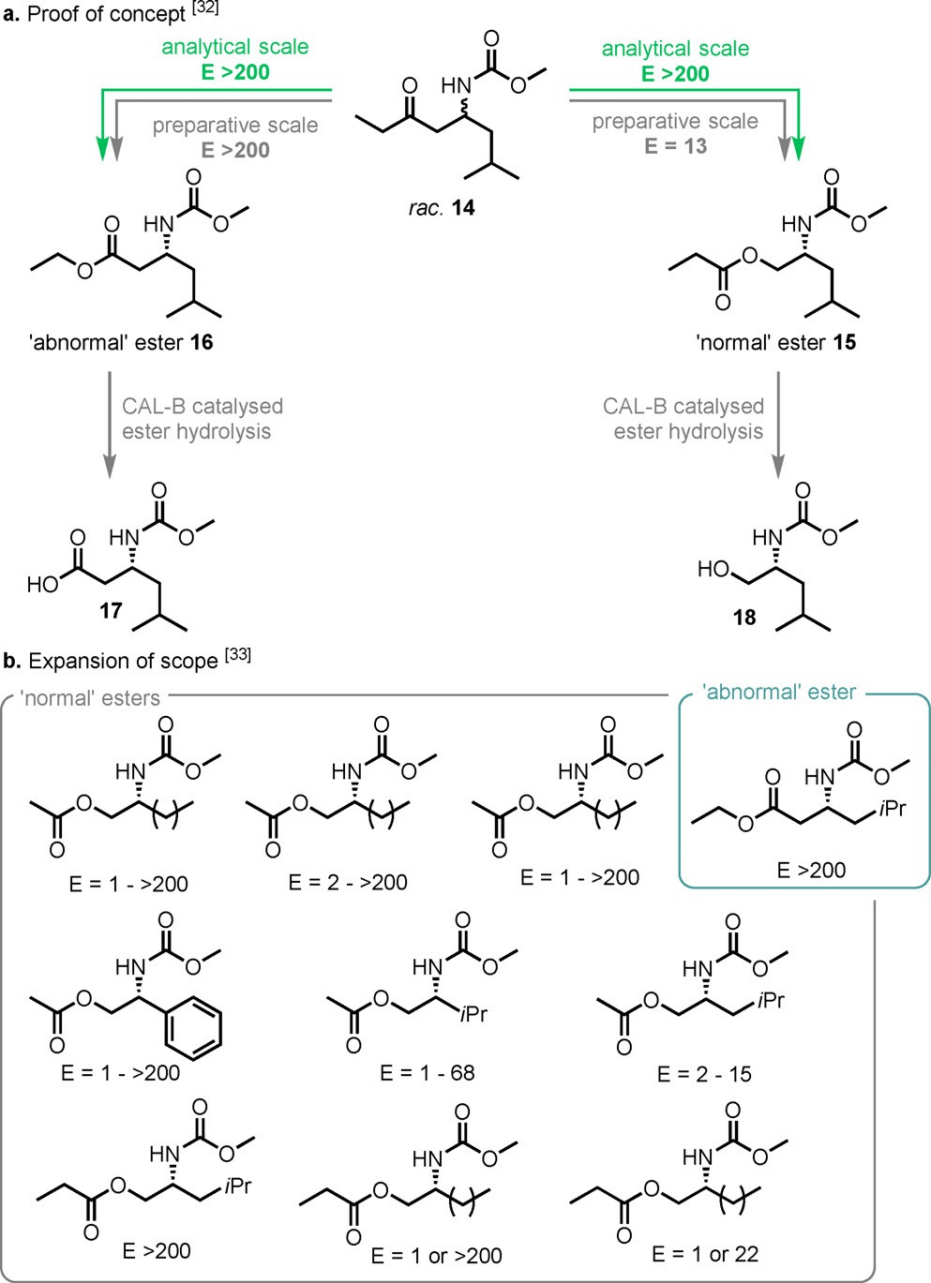
a) Initial report and b) further expansion of the scope of tolerated N‐protected β‐amino ketones to the corresponding “normal” or “abnormal” esters. Ten BVMOs from differing bacterial origins were tested (CHMO_Acineto_, CHMO_Arthro_, CHMO_Brachy_, CHMO_Xantho_, CHMO_Rhodo1_, CHMO_Rhodo2_, CHMO_Brevil_, CDMO, HAPMO_ACB_, and PAMO) with the highest E value reported here; see ref. [33] for full reported E values for each BVMO. Where the E number is reported only as *E*>200, either one or all the BVMOs with reported activity gave *E*>200.

The focus of biocatalytic BV KR research has predominantly been on cyclic and bicyclic substrates, with relatively few reported examples of linear aliphatic ketones as accepted substrates for BVMOs. In one example using N‐protected β‐amino ketone **14**, four BVMOs tested were all able to produce both the “normal” **15** and “abnormal” **16** esters with excellent E values (Scheme 4).^[32]^ After hydrolysis of the ester products with *Candida antarctica* lipase B (CAL‐B), high‐value enantioenriched β‐amino acid **17** and β‐amino alcohol **18** were obtained. Soon after this initial report, a more comprehensive study was published using ten BVMOs from varying bacterial origins, in which a wide range of aryl‐ and alkyl‐substituted ketones were converted, with E values >200.^[33]^

BVMOs are versatile enzymes whose catalytic activity can be employed to synthesise valuable heteroatom‐bearing chiral building blocks by non‐BV processes. For example, they are known to catalyse the oxidation of a wide range of heteroatoms including nitrogen, selenium, boron, phosphorus, and sulfur.^[34]^ Codexis used this reactivity in their large–scale synthesis of the chiral sulfoxide Esomeprazole, using an engineered BVMO to circumvent problems associated with the previously used Kagan–Sharpless–Pitchen type oxidation.^[35]^ There are relatively few examples here of KR as most reports focus on the oxidation of achiral substrates; however, there are reported cases of the OKR of racemic sulfoxides, boronates, and selenides.

**Scheme 5:**
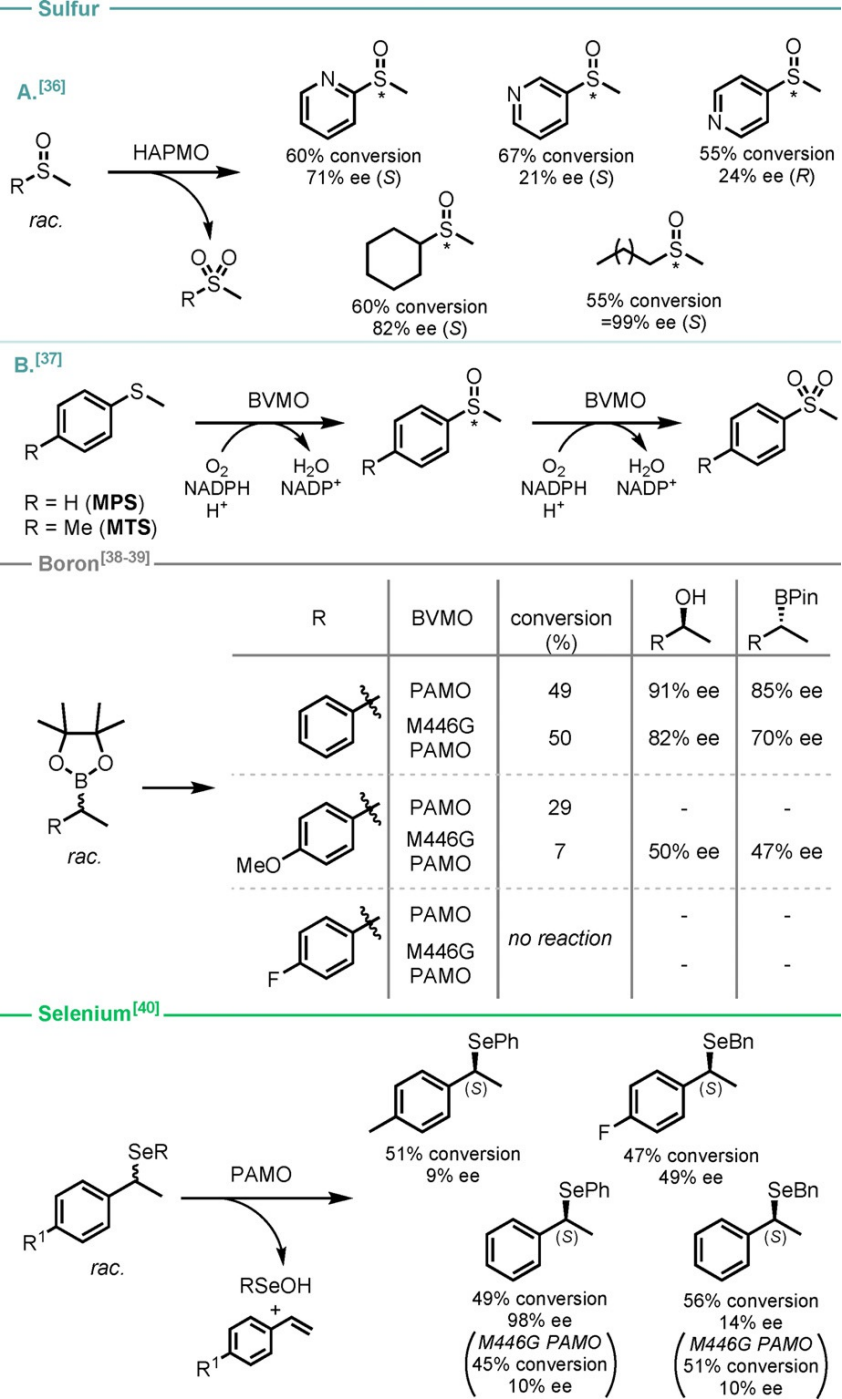
BVMO‐catalysed OKR of heteroatom‐containing substrates.

A *p*‐hydroxyacetophenone monooxygenase (HAPMO) was reported to catalyse the oxidation of racemic sulfoxides to sulfones in an enantioselective manner, leaving enantioenriched unreacted sulfoxides.^[36]^ Alkyl substituents were found to be better tolerated than aryl; however, high conversions and short reaction times (0.5–5.0 h) were achieved with all substrates (Scheme 5). Bornscheuer identified eight new monooxygenases from the eukaryote *Yarrowia lipolytica*, two of which were able to accept both sulfides and sulfoxides as substrates.^[37]^ The authors reported high enantioselectivities for the asymmetric oxidation of sulfides (methyl phenyl sulfide (MPS), methyl *p*‐tolyl sulfide (MTS), and l‐methionine) to the corresponding sulfoxides with multiple variants. Further efficient oxidation to the corresponding sulfones was possible; however, there was poor discrimination between sulfoxide enantiomers resulting in a low degree of KR.

Despite poor reported E values for the oxidation of phenylethylboronates, catalysed by either PAMO^[38]^ or its M446G mutant,^[39]^ this transformation produces two valuable products: the secondary alcohol and the unprocessed boronate, both with high optical purity.

Selected BVMOs were shown to catalyse the oxidation of secondary benzylic selenides to the corresponding enantioenriched selenoxides.^[40]^ The resulting selenoxides undergo β‐elimination, yielding styrene‐type structures as an unwanted side‐product, leaving the unreacted (*S*)‐selenide enantioenriched.

#### 2.1.2 Modes of Kinetic Resolution

As illustrated previously (Figure 2), KR can proceed via enantioselective and enantiodivergent pathways or, if racemisation of the substrate is possible, via DKR.

**Scheme 6:**
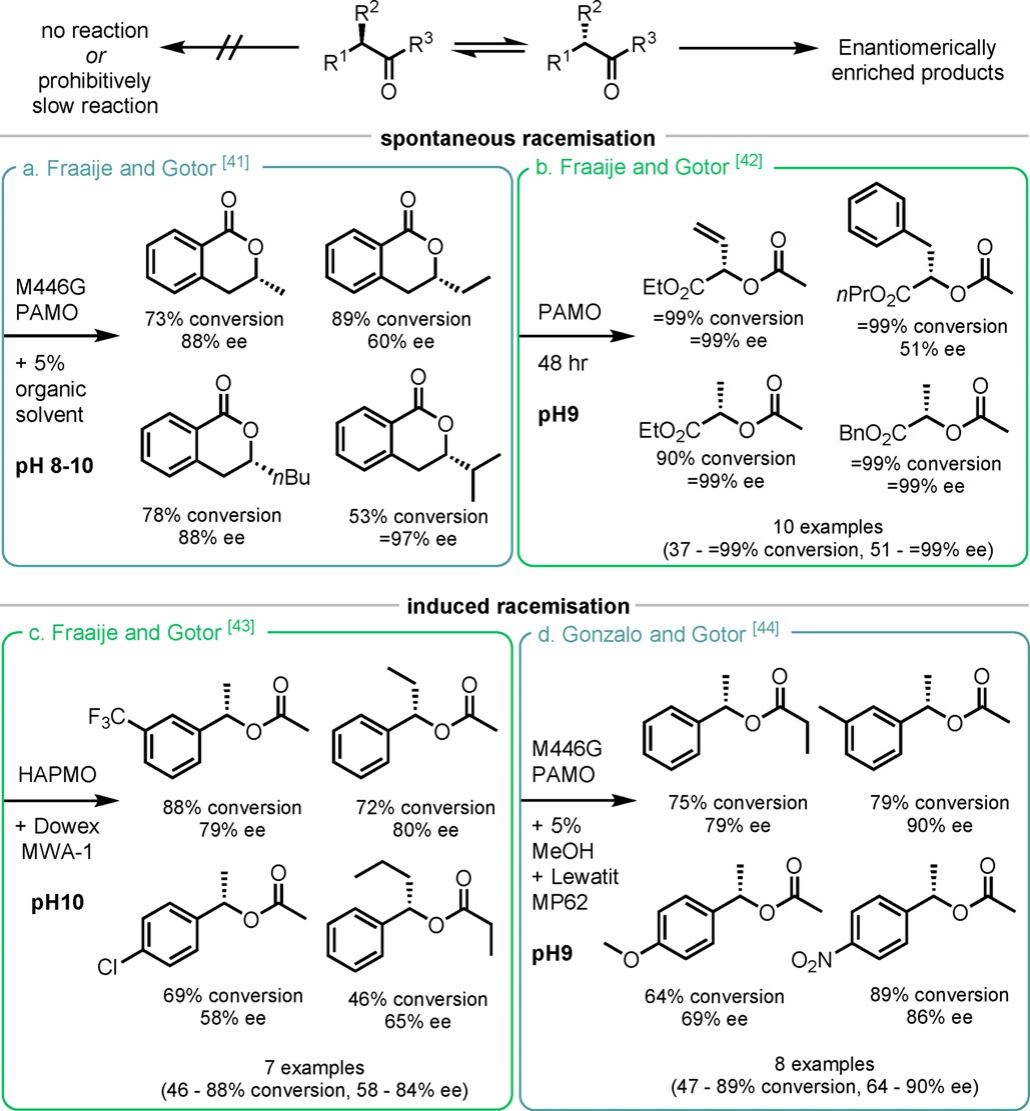
Methods of substrate racemisation for DKR. Selected examples shown for refs. [42, 43, 44]; all four products shown for ref. [41].

Gotor and Fraaije took advantage of acidic substrates to achieve DKR. The commonly used M446G mutant of PAMO produced a range of 3‐alkyl‐3,4‐dihydroisocoumarins with high enantioselectivities (Scheme 6 a), although the remaining α‐alkyl indanones substrates showed low *ee* (all <35 %).^[41]^ This class of substrates readily racemise under the mildly basic reaction conditions (pH 8–10) and it was found that the addition of small amounts of organic solvent (MeOH or hexane) improved enzyme activity or selectivity. The authors extended this methodology using two linear‐ketone‐converting BVMOs, PAMO and HAPMO, to convert a range of α‐substituted β‐ketoesters, which spontaneously racemise at pH 9, to the corresponding diesters (Scheme 6 b). These products are easily hydrolysed via chemical methods to produce valuable enantioenriched α‐hydroxy esters.^[42]^

The authors intended to extend this concept to include acyclic benzyl ketones; however, for this class of compounds, the addition of an anion‐exchange resin was required to promote racemisation.^[43]^ It was found that less basic resins achieved substrate racemisation without adversely affecting the activity of the isolated HAPMO, albeit with prolonged reaction times (selected examples, Scheme 6 c). Replicating previously successful conditions, using the mutant M446G PAMO and a small amount of hydrophilic solvent, the same authors were able to further improve the performance of both the standard and dynamic kinetic resolution for these substrates (Scheme 6 d).^[44]^ The activity of BVMOs is generally inhibited by a high concentration of substrate and product but Bornscheuer and co‐workers were able to overcome this issue by using a hydrophobic adsorbent resin.^[45]^ This allowed both the slow release of substrate and the adsorption of product, such that the HAPMO‐catalysed KR of these substrates proceeded with E values >100 on a synthetically useful scale (5 mmol).

**Scheme 7:**
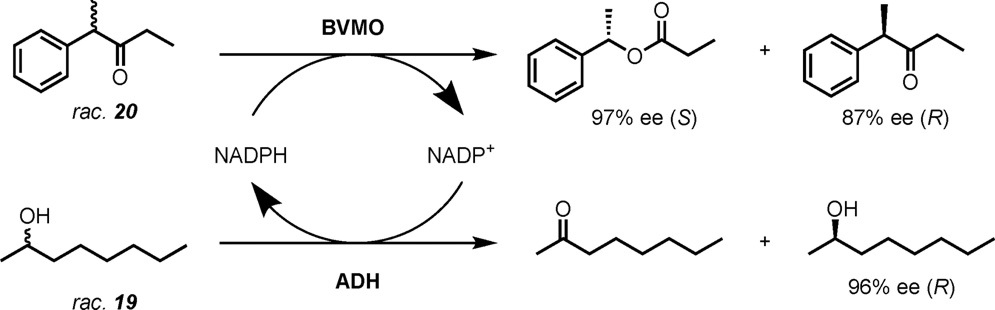
PIKAT of racemic ketone **19** and racemic alcohol **18**, achieving high *ee* and an E value >200 for the desired ester.

A significant disadvantage for BVMO biotransformations compared to chemical methods of oxidation is the requirement for stoichiometric amounts of the expensive cofactor NADPH and for large scale applications it is essential to develop effective nicotinamide cofactor regeneration systems.^[46]^ Parallel interconnected kinetic asymmetric transformations (PIKAT) allow an ADH to regenerate NADPH through the selective oxidation of secondary alcohol **19**, leaving the unreacted enantiomer of alcohol **19** with high optical purity.^[47]^ The BVMO‐mediated kinetic resolution of ketone **20** shown in Scheme 7 occurs with an excellent E value (*E*>200) leading to the concurrent production of three valuable chiral compounds.

#### 2.1.3 Targeted Applications

**Scheme 8:**
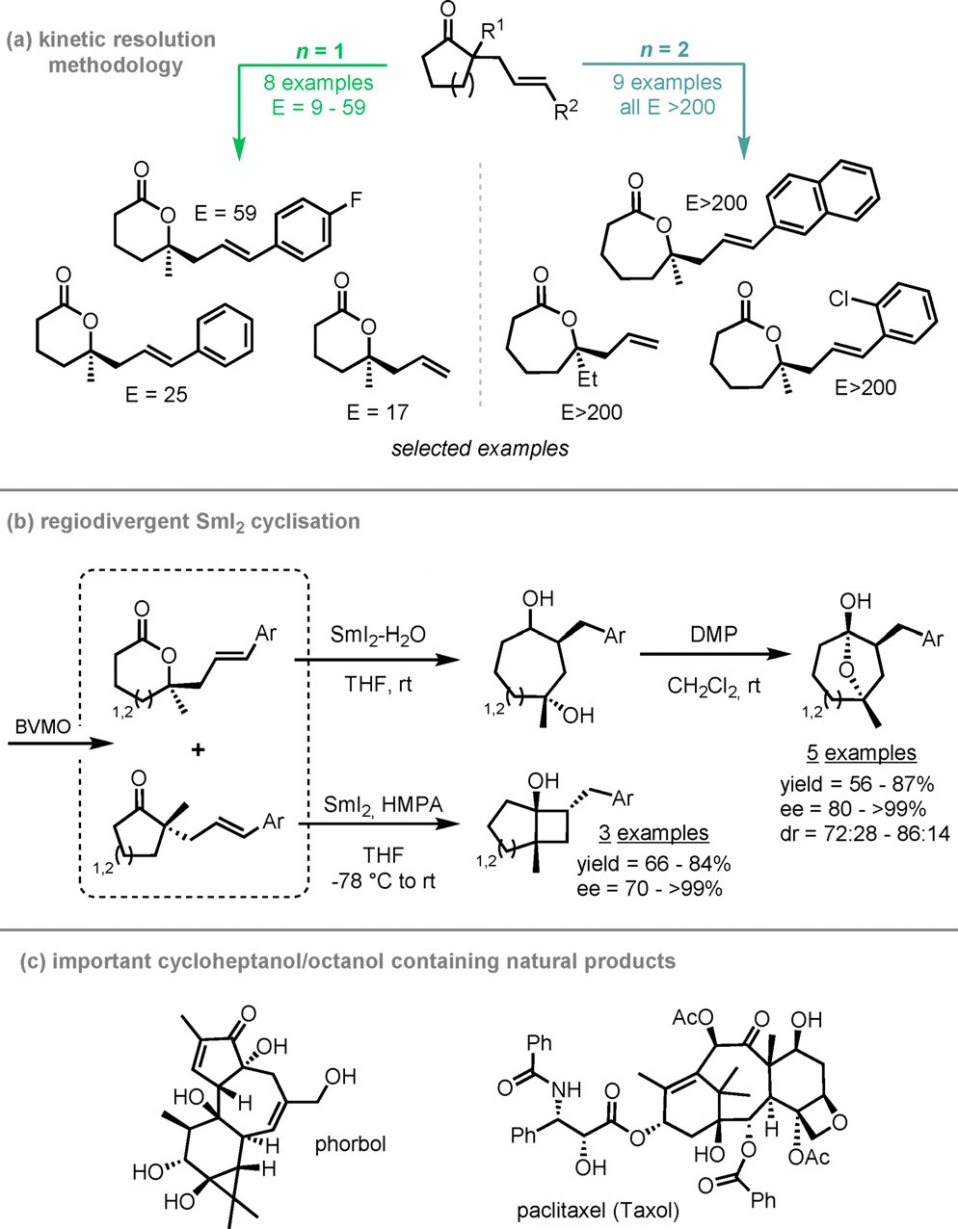
a) CHMO_Acineto_‐catalysed BV KR of five‐ and six‐ membered cyclic ketones (selected product examples shown); b) extension of the methodology to SmI_2_‐mediated reductive cyclisation; c) complex natural products containing cycloheptanol and cyclooctanol core structures.

OKR using BVMOs has many advantages in complex target synthesis, both for natural product synthesis and for the production of other challenging compound classes. Turner and Procter used a CHMO from *Acinetobacter calcoaceticus* to catalyse the KR of a wide range of five‐ and six‐membered cyclic ketones to lactones with excellent E values (Scheme 8 a).^[48]^ This was the first report of a BVMO kinetic resolution of α,α‐dialkyl cyclic ketones, with previous work in this area focusing on α‐monoalkyl cyclic ketones (cf. Scheme 3). The authors develop the methodology further in an enantiodivergent process (Scheme 8 b); in this, both lactone and remaining ketone proved to be suitable substrates for a SmI_2_‐mediated reductive cyclisation, producing enantioenriched bridged or fused cycloalkanols which feature as key core structures in a range of complex natural products (examples in Scheme 8 c).

**Scheme 9:**
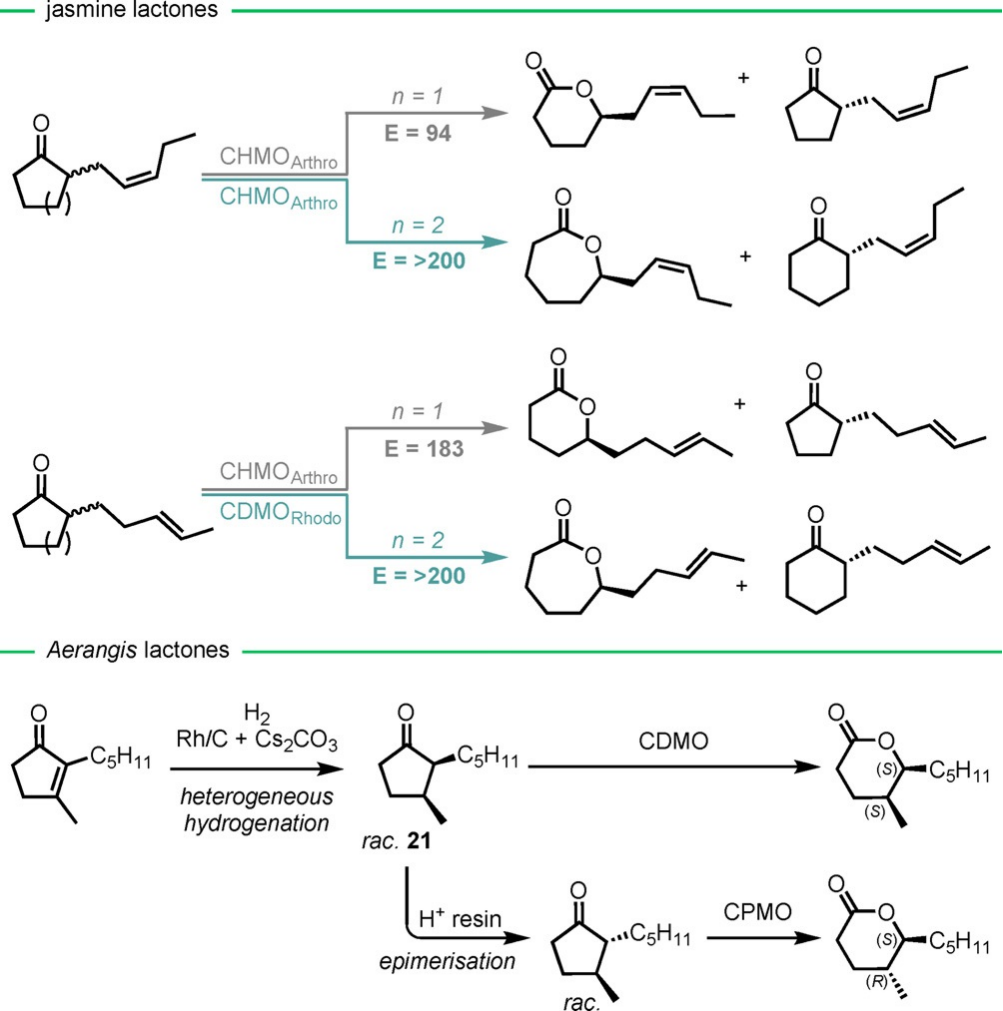
BVMO biotransformations for the preparation of enantioenriched lactones.

The Mihovilovic group obtained naturally occurring fragrance lactones by whole‐cell BVMO biotransformations on a preparative scale (100–150 mg).^[49]^ In this report excellent E values were achieved in both analytical‐ and preparative‐scale reactions of the six‐membered‐ring jasmine lactones and their caprolactone homologues (Scheme 9). This was extended by combining continuous‐flow hydrogenation with a cyclododecanone monooxygenase catalysed Baeyer–Villiger oxidation (BVOx) in a single‐operation chemoenzymatic sequence to produce *Aerangis* lactones.^[50]^ The kinetic resolution of *cis*‐**21** occurred with excellent selectivity (*E*>200) and is the first reported case of a diastereoselective BVOx to produce the natural diastereomer of an *Aerangis* lactone as the only product (>99 % de).

**Scheme 10:**
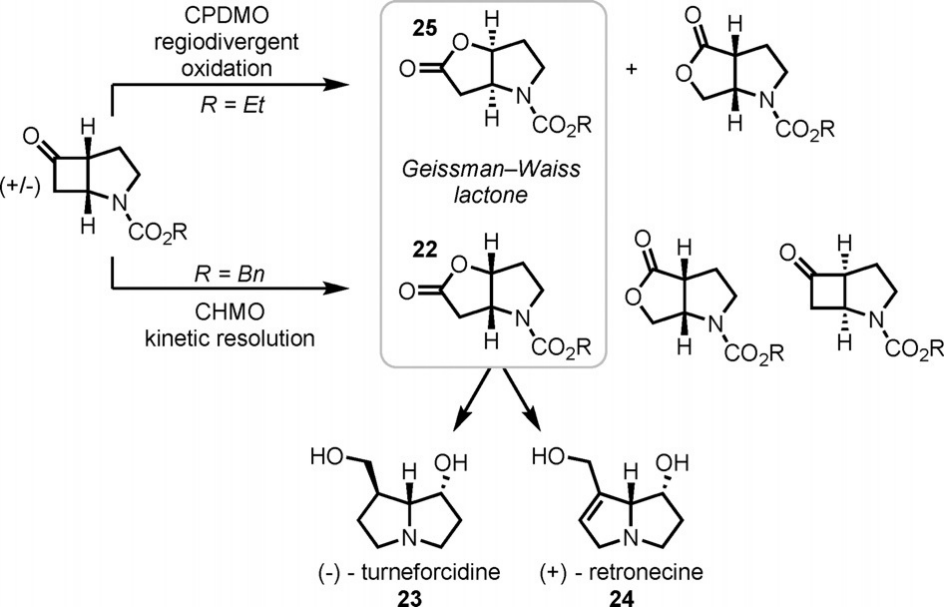
Access to both enantiomers of the Geissman–Waiss lactone as intermediates in pyrrolizidine synthesis.

The Geissman–Waiss lactone **22** is a key intermediate in the total synthesis of many pyrrolizidine alkaloids, including (−)‐turneforcidine (**23**) and (+)‐retronecine (**24**) (Scheme 10).^[51]^ Inspired by earlier work from Alphand on the CHMO‐catalysed kinetic resolution of this intermediate,^[52]^ Mihovilovic reported a regiodivergent cyclopentadecanone monooxygenase (CPDMO)‐catalysed oxidation producing the lactone **25**, which leads to the non‐natural enantiomers of these natural products.^[53]^

**Scheme 11:**
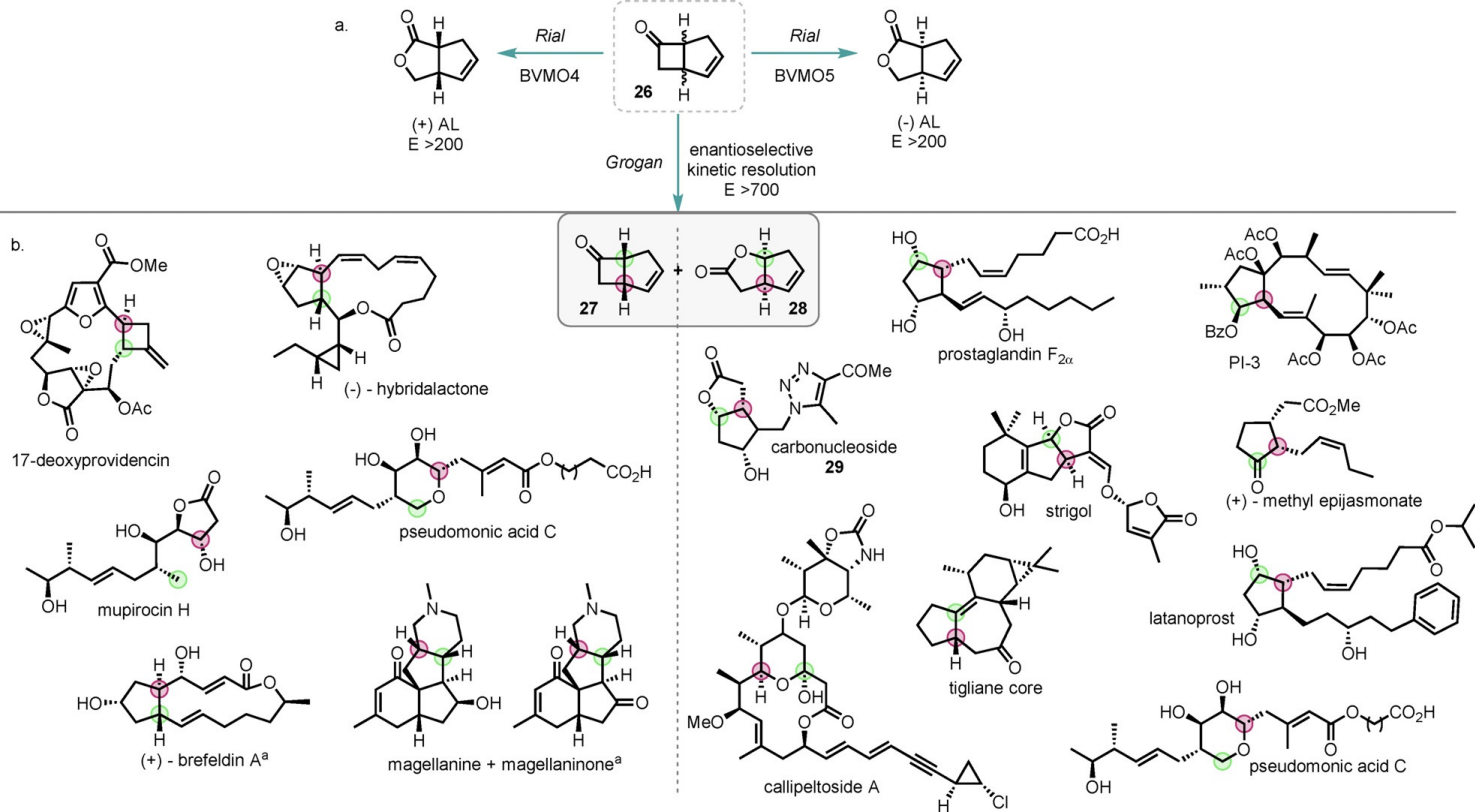
a) Notable methodology examples of whole‐cell biotransformations of the fused bicyclic ketone **26**; b) natural products whose total synthesis proceeds through these key chiral building blocks. The stereogenic centres produced as a result of the BVMO kinetic resolution are highlighted in the final natural product structure. [a] Using the (1*R*,5*S*) enantiomer.

Fused bicyclic ring systems are excellent substrates for BVMOs due partially to the release of ring strain, and there are many examples of the oxidation of racemic fused cyclobutanones.^[54]^ The cyclic ketone **26** (Scheme 11) is an exceptional model system to study BVMO OKR because its structural features lead to interesting and diverse products through the enantio‐ and regioselective oxidative transformation. Through genome mining, Opperman found four phylogenetically distinct new BVMOs from the fungus *Aspergillus flavus*, three of which were able to catalyse the oxidation of ketone **26** in up to 99 % *ee*.^[55]^ Rial also used genome mining to identify seven new BVMOs, two of which were able to provide the abnormal lactones (AL) selectively from the model bicyclic ketone **26** (Scheme 11 a).^[56]^ Two of the new BVMOs gave an effective KR, each yielding one enantiomer of the abnormal lactone selectively with *E*>200. This is the first report of a prokaryotic BVMO giving the abnormal (+)‐lactone selectively.

This bicyclic ketone is an excellent substrate for KR, with an impressive whole‐cell enantioselective KR with *E*>700 reported by Grogan.^[57]^ This important research highlights the ability of biotransformations to provide valuable enantioenriched intermediates on an industrially relevant scale. These compounds are key chiral intermediates in a plethora of reported natural product syntheses (Scheme 11 b). Thus, the enantiomerically enriched lactone **28** has been used to prepare the Corey lactone diol (leading to the synthesis of a vast selection of prostaglandins,^[58]^ including the shown prostaglandin F2α^[59]^ and latanoprost^[60]^), jatrophane diterpenes such as PI‐3,^[61]^ the tigliane ring system,^[62]^ callipeltoside A,^[63]^ strigol,^[64]^ methyl epijasmonate,^[65]^ pseudomonic acid C,^[66]^ carbonucleoside **29**,^[67]^ and various antivirals; the ketone **27** finds application in the synthesis of mupirocin H,^[68]^ brefeldin A,^[69]^ magellanine and magellaninone,^[70]^ pseudomonic acid C,^[71]^ 17‐deoxyprovidencin,^[72]^ and hybridalactone.^[73]^

### 2.2 Epoxidation

**Scheme 12:**
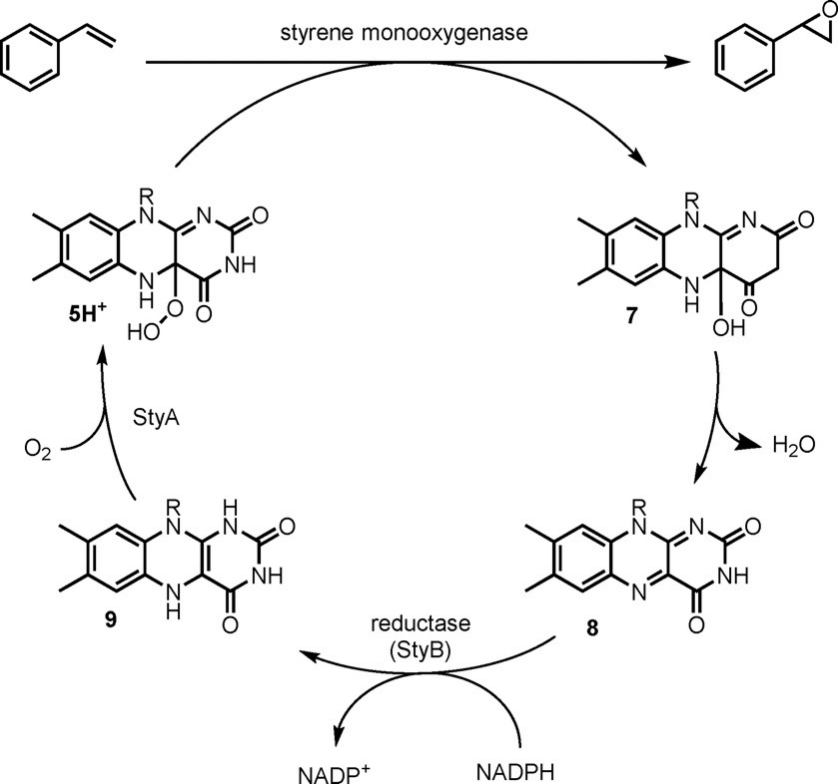
Catalytic cycle of styrene monooxygenase.

Epoxidation by chemical means has long been established as an effective method for the KR of racemic olefin substrates,^[74]^ including pioneering work by Sharpless,^[75]^ Jacobsen,^[76]^ Katsuki,^[77]^ and Shi.^[78]^ Although there has been considerable research into biocatalytic asymmetric epoxidations,^[79]^ there are relatively few examples that extend this methodology to achieve KR. Styrene monooxygenases are commonly used biocatalysts for this transformation. Similar to BVMO they are flavoproteins, but consist of two components. The reductase StyB uses NADPH to catalyse the reduction of flavin **8** to FADH_2_ **9**, which is then transferred to the oxidase StyA to undergo a reaction with molecular oxygen to produce the peroxy‐flavin **5H^+^** as the active epoxidising agent (Scheme 12). The system is often paired with a regeneration system to re‐form NADPH from NADP^+^.

**Scheme 13:**
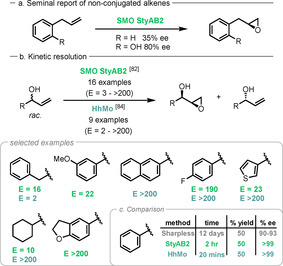
Initial report of SMO tolerance of nonconjugated alkenes for asymmetric epoxidation by Wu. Selected examples of the kinetic resolution of allylic alcohols by SMO StyAB2 and HhMo.

Early literature on styrene monooxygenases (SMOs) focused solely on aromatic sulfides and alkenes, such as styrene derivatives; however, in 2010 Wu reported the first asymmetric epoxidation of nonconjugated alkenes by the novel SMO StyAB2,^[80]^ thereby opening up the potential for a much wider substrate scope.^[81]^ It was found that the presence of a hydroxyl group in the substrate significantly improved the *ee* (Scheme 13 a) and enabled application to the kinetic resolution of allylic secondary alcohols (Scheme 13 b).^[82]^ Two new bacterial SMOs, PaSMO and MlSMO, were identified through genome mining of *Paraglaciecola agarilytica* and *Marinobacterium litorale*, respectively. These enzymes showed improved activity and selectivity in whole‐cell biotransformations for asymmetric epoxidation; however, no E values were reported for the allylic alcohol tested.^[83]^

Using a reconstructed ancestral protein from six known SMO enzyme sequences, Chen used genome mining to identify a new monooxygenase from *Herbaspirillum huttiense* (HhMo) able to catalyse the epoxidation of nonconjugated terminal alkenes.^[84]^ Excellent enantioselectivities were achieved (>99 % *ee* for all examples) and far superior results were obtained by comparison with traditional chemical methods such as the Sharpless epoxidation (Scheme 13 c). Importantly, this work is the first to report tolerance of aliphatic substrates in a monooxygenase‐catalysed epoxidation.

## 3 Kinetic Resolution via C–H activation

Direct functionalisation of unactivated C−H bonds has emerged as one of the most important focuses in modern synthetic organic chemistry,^[85]^ with many elegant applications to KR.^[86]^ Among the many advantages in employing enzymes in synthesis is the ability to functionalise positions in a compound which are inaccessible or inactive towards chemical reagents; this ability has been employed in many chemoenzymatic total synthesis applications. Despite this, enzymes have been largely overlooked as reagents to achieve the deliberate resolution of racemic intermediates via C–H activation; frequently, studies only utilised the native substrate in conjunction with the known corresponding enzyme from its biosynthetic pathway.

**Scheme 14:**
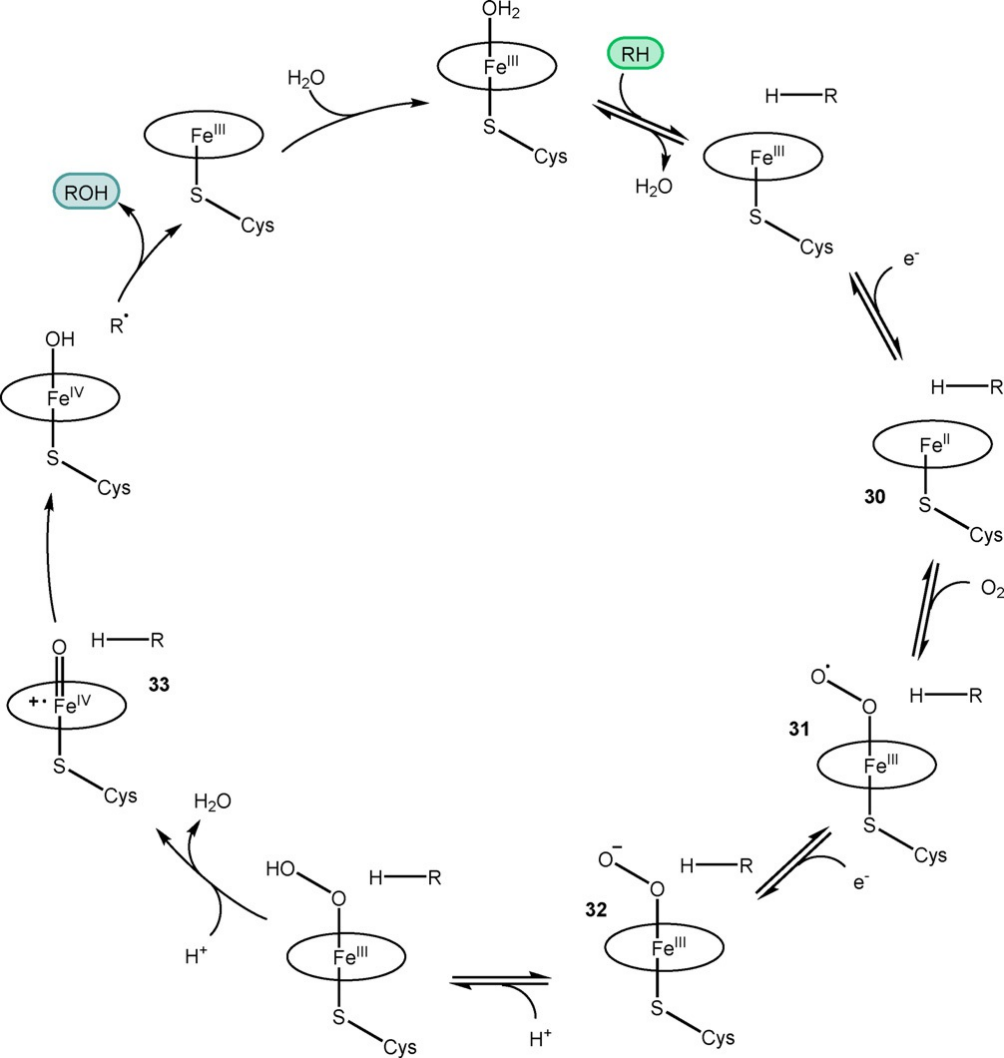
Cytochrome P450 catalytic cycle.

One of the most important enzyme classes for oxidation via C–H activation is the hemoprotein cytochrome P450s.^[87]^ The catalytic cycle initiates with substrate binding and displacement of a water molecule, inducing an active site conformational change (Scheme 14). Electron transfer from the associated P450 reductase domain produces an Fe^II^ species **30** which reacts with molecular oxygen to produce **31** which, after another electron transfer, forms the ferric peroxy complex **32**. Two subsequent protonations, along with a loss of water and heterolytic cleavage of the O−O bond, results in the formation of the highly active ferryl Compound I species **33**, which is the key intermediate for substrate oxidation. Release of oxidised substrate and binding of a water molecule completes the catalytic cycle.

**Scheme 15:**
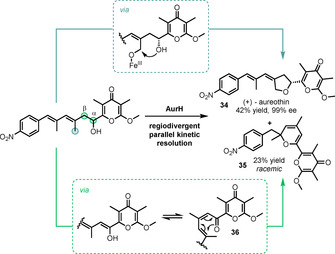
P450‐mediated regiodivergent parallel KR.

Hertweck and De Paolis reported the first parallel kinetic resolution using the P450 monooxygenase AurH in their synthesis of aureothin (**34**),^[88]^ expanding on Hertweck's earlier report of the first chemoenzymatic total synthesis of this natural product.^[89]^ This unprecedented regioselective C−H bond oxidation, and subsequent cyclisation, installed the tetrahydrofuran ring in the final step, thereby circumventing issues such as olefin isomerisation and stereocentre epimerisation that had complicated previously reported syntheses.^[90]^ In this transformation, the natural product is produced through selective allylic hydroxylation of one enantiomer and cyclisation with both high yield and enantioselectivity, recovery of some enantioenriched starting material (19 % yield, 85 % *ee*), and production of the unexpected racemic pyran **35** (Scheme 15). The pyran is proposed to arise through oxidation at α‐ or β‐ positions (highlighted in green) of the other enantiomer, then elimination, tautomerisation, and 6π‐electrocyclisation of the intermediate triene **36** to yield pyran **35** in 23 % yield.

**Scheme 16:**
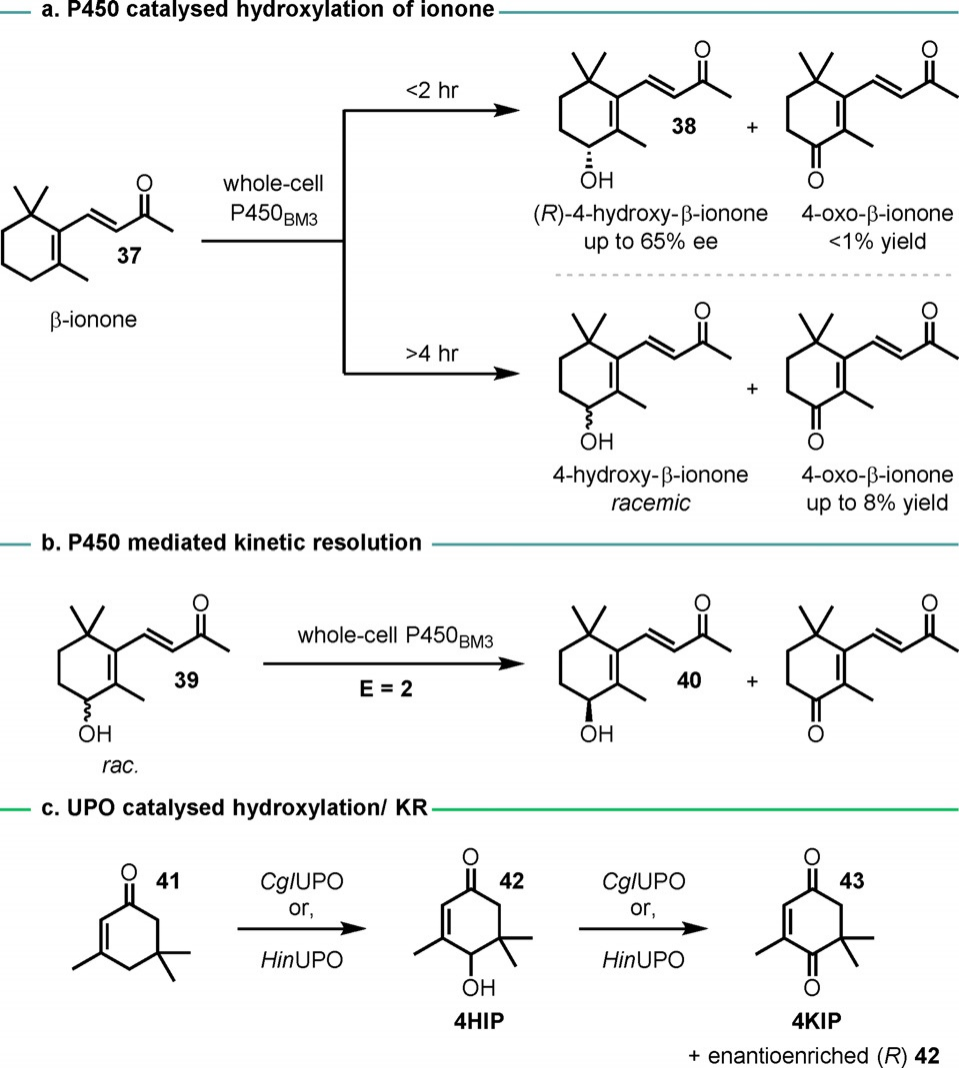
P450_BM3_‐ and UPO‐catalysed OKR.

Lütz reported a KR while investigating the enantioselective whole‐cell oxidation of β‐ionone (**37**) using P450_BM3_ enzymes.^[91]^ A moderate *ee* was established for (*R*)‐4‐hydroxy‐β‐ionone **38** produced within short reaction times; loss of enantiomeric purity occurred in prolonged reactions due to over‐oxidation to the corresponding ketone (Scheme 16 a). This suggests that the (*R*)‐hydroxylated product **38** is the preferred enantiomer in the oxidation to 4‐oxo‐β‐ionone, and this was confirmed in an experiment with the oxidation of the racemic alcohol **39** as starting material which returned enantioenriched (*S*)‐alcohol **40** (Scheme 16 b). The reported E value is low (*E*=2) but the transformation represents an important proof of principle that P450 monooxygenases are capable of achieving KR of racemic alcohols.

Similarly, Martínez and Gutiérrez found unspecific peroxygenases (UPOs) that were able to catalyse the enantioselective hydroxylation of isophorone (**41**) (Scheme 16 c).^[92]^ The authors found that over‐oxidation to 4KIP **43** led to a reduction of the enantiomeric purity of the 4HIP product **42** when UPOs from the ascomycetes fungi *Chaetomium globosum* (*CgI*UPO) and *Humicola insolens* (*Hin*UPO) were used. It was found that the (*S*)‐enantiomer of 4HIP was oxidised preferentially, and KR of racemic 4HIP could achieve 99–100 % *ee*.

**Scheme 17:**
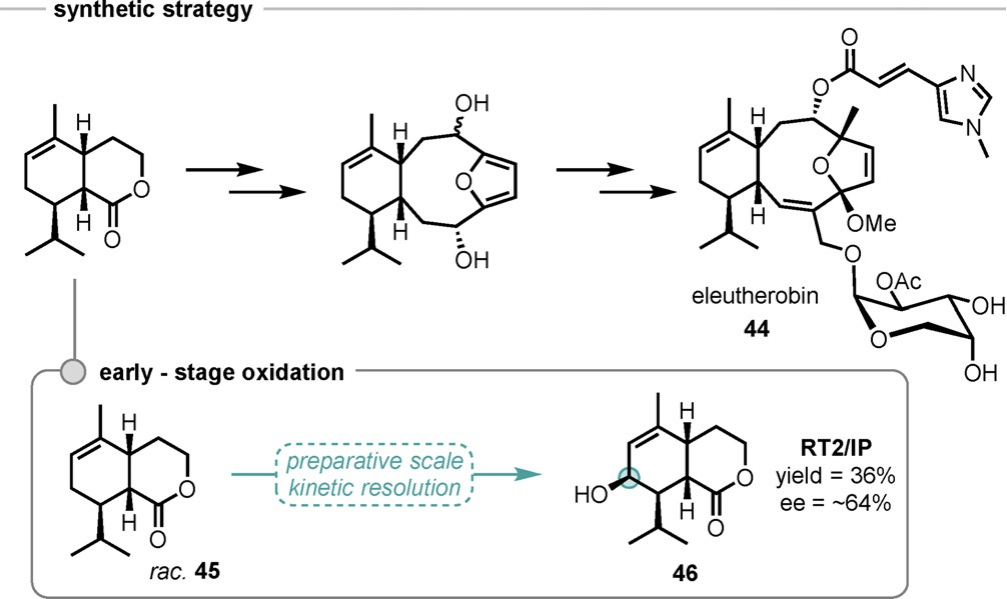
Chemoenzymatic synthetic strategy toward eleutherobin **44** and hydroxylated analogues, and discovery of OKR by P450_BM3_‐catalysed hydroxylation of an early intermediate **45**.

Robertson and Wong reported the kinetic resolution, by P450_BM3_ mutants, of intermediates in the formal synthesis of eleutherobin (**44**) and oxidised analogues.^[93]^ As part of this study, lactone **45** was screened against a panel of 24 mutants to give a diverse set of hydroxylated products (Scheme 17). In a small‐scale preparative reaction (42 μmol), using mutant RT2/IP, alcohol **46** was obtained in 64 % *ee* and 36 % yield.

**Scheme 18:**
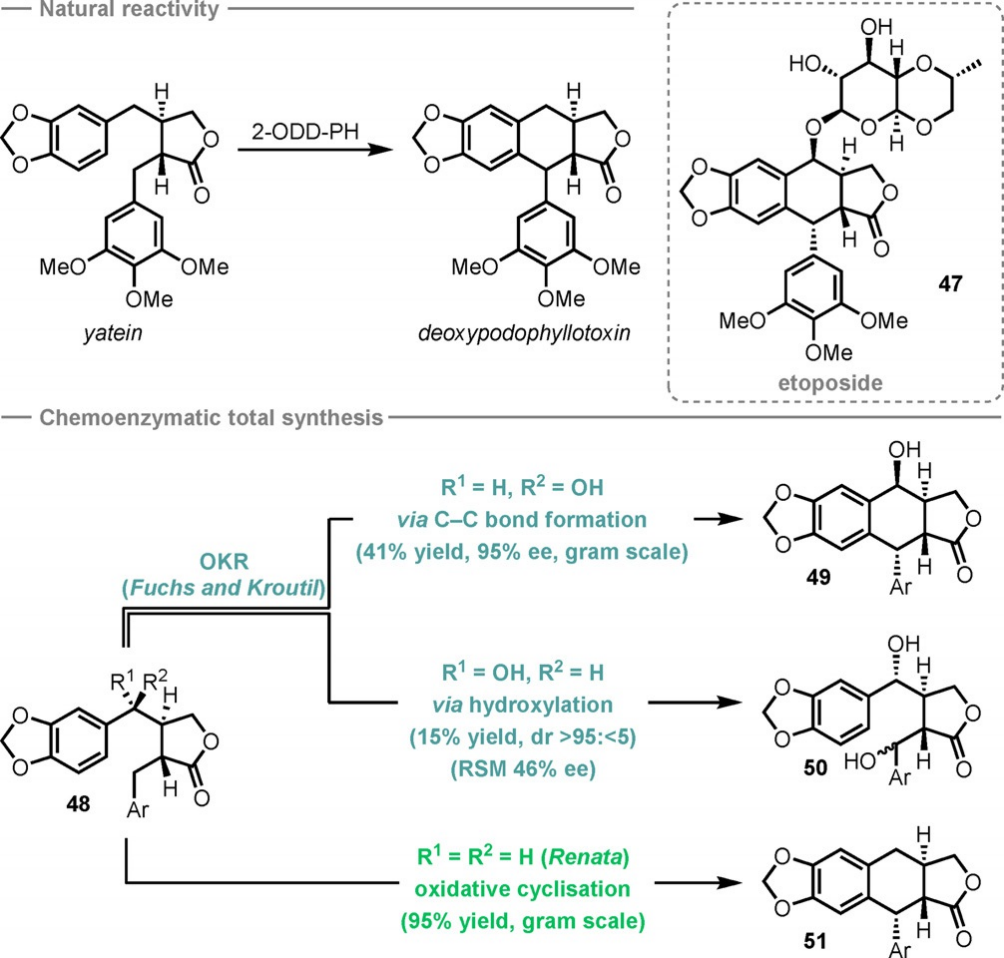
The role of 2‐ODD‐PH in the biosynthesis of etoposide (**47**) and the reported total syntheses of podophyllotoxin (**49**). Ar=3,4,5‐trimethoxyphenyl.

Soon after the publication of a report on the role of a 2‐oxoglutarate‐dependent dioxygenase (2‐ODD) in a key step of the biosynthetic pathway of etoposide (**47**),^[94]^ two groups described chemoenzymatic routes to podophyllotoxin (**49**) patterned on this biosynthetic step. In the first, Kroutil and Fuchs reported an impressive large‐scale enzymatic kinetic resolution of *rac*‐**48** via enantioselective ring‐closing C−C bond formation.^[95]^ Although this transformation was shown not to proceed through a hydroxylated intermediate, the authors reported initial results in which one substrate enantiomer was selectively hydroxylated to **50**, leaving enantioenriched starting material behind. The stereochemistry at the benzylic alcohol centre in the substrate determines the reactivity (Scheme 18), leading to the proposal that substrate positioning within the 2‐ODD‐PH active site is crucial. In the second report, Renata's group used enantiopure starting material **48** in a gram‐scale oxidative cyclisation, achieving excellent yields of **51** and installing the hydroxy functionality at a later stage.^[96]^ Both groups prepared a range of podophyllotoxin‐related structures to test the 2‐ODD‐PH substrate scope and reported a range of both hydroxylated and cyclised products.

## 4 Summary and Outlook

This Minireview highlights the considerable research focus on BVMOs, and the body of literature is much larger than for other biocatalytic oxidative kinetic resolution transformations such as epoxidation or hydroxylation via C–H activation. Even so, there remains, in general, a lack of direct application of the results to synthesis, whether for natural product chemistry or use in industrial routes to, for example, pharmaceuticals. Biotechnologists have focused on enzyme development, leading to impressive advancements in enzyme stability and scalability, as well as discovering transformations that produce interesting molecules in enantioenriched form with synthetically useful conversions and high selectivity. Many of these molecules have been employed by synthetic chemists in routes to natural product intermediates or privileged structure classes; more often than not, however, the two worlds have not intersected, with the chiral intermediates actually taken on in multistep synthesis not necessarily being sourced by biocatalytic oxidative KR.

There is, however, a growing number of academic and industrial groups who conduct research at the interdisciplinary interface of biocatalysis and chemical synthesis, in addition to those willing to collaborate, allowing the transfer of knowledge and skills. There are huge opportunities for methodological and technological advances in biocatalysis to influence organic synthesis profoundly,^[97]^ and vice versa. We are confident that the field will continue to flourish with the development of elegant new strategies and methods arising from seamless applications of both chemical and enzymatic transformations in complex target syntheses.^[98]^

## Conflict of interest

The authors declare no conflict of interest.

## Biographical Information

*Lucy Harwood received her MSci degree in Chemistry from Queen's University*, *Belfast, completing her final year research project on the use of sigmatropic rearrangements in synthesis in the group of Prof. P. Stevenson. She is currently conducting her doctoral studies at the University of Oxford in the groups of Prof. J. Robertson and Prof. L.‐L. Wong developing mutant cytochrome P450s as general oxidation catalysts*.

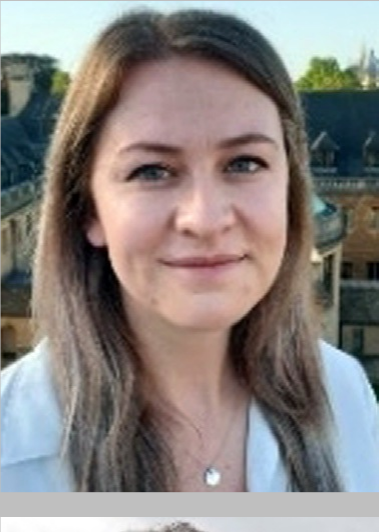

## Biographical Information

*Luet Lok Wong is Professor of Chemistry at the University of Oxford within the Department of Chemistry, and the Jennifer Clare Green Fellow and Tutor in Inorganic Chemistry at St Hugh's College, Oxford. He received his DPhil (1987) in organometallic chemistry under Prof. Malcolm L. H. Green at Oxford. He conducted postdoctoral research for two years at Wolfson College, Oxford, followed by two years at the California Institute of Technology working with Harry B. Gray. He returned to Oxford in 1991 to initiate a research programme on discovering and engineering cytochrome P450 enzymes for applications in synthesis and synthetic biology*.

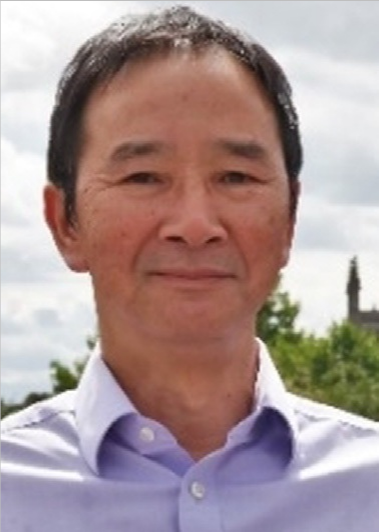

## Biographical Information

*Jeremy Robertson is Professor of Chemistry at the University of Oxford within the Department of Chemistry, and Fellow and Tutor in Organic Chemistry at Brasenose College, Oxford. He received his DPhil (1990) under Sir Jack Baldwin at Oxford and then spent two years at Columbia University working with Gilbert Stork. Upon returning to the UK in 1992, he initiated a research programme to develop new synthetic methods within the context of natural products and biologically active compounds.Together with Luet Wong, he manages a research team in China at the Oxford Suzhou Centre for Advanced Research (OSCAR) to explore applications of engineered P450 mutants as general oxidation catalysts*.